# Construction of an overexpression library for *Mycobacterium tuberculosis*

**DOI:** 10.1093/biomethods/bpy009

**Published:** 2018-08-20

**Authors:** Eduard Melief, Rachel Kokoczka, Megan Files, Mai Ann Bailey, Torey Alling, Hongye Li, James Ahn, Ayesha Misquith, Aaron Korkegian, David Roberts, James Sacchettini, Tanya Parish

**Affiliations:** 1 TB Discovery Research, Infectious Disease Research Institute, Seattle, WA, USA; 2 Department of Biochemistry and Biophysics, Texas A&M University, College Station, TX, USA

## Abstract

There is a pressing need to develop novel anti-tubercular drugs. High-throughput phenotypic screening yields chemical series that inhibit bacterial growth. Target identification for such series is challenging, but necessary for optimization of target engagement and the development of series into clinical drugs. We constructed a library of recombinant *Mycobacterium tuberculosis* strains each expressing a single protein from an inducible promoter as a tool for target identification. The library of 1733 clones was arrayed in 96-well plates for rapid screening and monitoring growth. The library contains the majority of the annotated essential genes as well as genes involved in cell wall and fatty acid biosynthesis, virulence factors, regulatory proteins, efflux, and respiration pathways. We evaluated the growth kinetics and plasmid stability over three passages for each clone in the library. We determined expression levels (mRNA and/or protein) in 396 selected clones. We screened the entire library and identified the Alr-expressing clone as the only recombinant strain, which grew in the presence of d-cycloserine (DCS). We confirmed that the Alr-expressing clone was resistant to DCS (7-fold shift in minimum inhibitory concentration). The library represents a new tool that can be used to screen for compound resistance and other phenotypes.

## Introduction

One-quarter of the global population is infected with *Mycobacterium tuberculosis*, the causative agent of tuberculosis (TB) [1, 2]. Due to the increasing prevalence of drug-resistant tuberculosis and an ageing arsenal of anti-tuberculosis drugs, there is a pressing and continuing need to develop novel, well-tolerated anti-tubercular drugs. Current TB drug discovery has focused on phenotypic screens against chemical libraries to identify compound scaffolds that inhibit bacterial growth [3, 4]. Treated as series, these compounds are optimized to minimize mammalian cytotoxicity while maintaining antibacterial activity and improving bioavailability. Identification of the target or metabolic pathway is crucial to drug discovery and development to improve on-target activity and specificity.

While genetic and biochemical methods have been applied successfully to determine intracellular targets in *M. tuberculosis* [5–16], identification of a target is often difficult and requires multiple approaches. For example, the isolation of resistant mutants may lead to target identification in about 20% of cases [5, 14]. Additional methods for target identification will therefore increase the potential to identify relevant targets or metabolic pathways, enhancing drug development. Target overexpression conferring resistance to antimycobacterial compounds has been used as a diagnostic or confirmatory assay once the target has been identified; in these cases, the target was previously predicted by other methods and overexpression was used to confirm the target [12, 17–22].

Overexpression libraries have been used in *Schizosaccaromyces* and *Saccharomyces*, covering 90 and 95% of each species ORF, respectively [23, 24]. In *Escherichia coli*, heterologous expression of genomic libraries from *Piriformospora indica* and *Marinobacter aquaeolei* identified genes conferring salt and terpene tolerance, respectively [25, 26]. Likewise, an inducibly expressed library of *E. coli* transcription factors was used to identify factors that mediated beta-lactam sensitivity in antibiotic-resistant *E. coli* [27]. In *M. tuberculosis*, systematic overexpression of transcription factors was used to derive a transcription factor interaction network [28].

In order to expand the methods available for target identification, we constructed a library of *M. tuberculosis* strains using an inducible expression system in which each clone expresses a single *M. tuberculosis* protein. We evaluated each clone for growth under induced and noninduced conditions and determined plasmid stability over several passages. We also monitored expression, either by protein or mRNA levels, for a subset of clones. The library was arrayed in 96-well plates for ease of use. We confirmed the functionality of the system by screening the library for resistance to d-cycloserine (DCS) and confirmed that the clone expressing *alr* was selected.

## Materials and methods

### *Mycobacterium tuberculosis* culture

*Mycobacterium tuberculosis* was grown in Middlebrook 7H9 medium supplemented with 0.05% w/v Tween 80 and 10% v/v oleic acid, albumen, dextrose, catalase (OADC) supplement (Becton Dickinson), or on Middlebrook 7H10 agar with 10% v/v OADC. Hygromycin (Hyg) was added to 100 µg/ml and anhydrotetracycline (ATc) to 150 ng/ml where required. For long-term storage, an equal volume of 50% w/v glycerol was added to 96-well plates and stored at -80°C.

### Library construction

The majority of the clones were constructed by polymerase chain reaction (PCR) amplification from H37Rv genomic deoxyribonucleic acid (DNA) using oligonucleotides incorporating the Gateway recombination sequences; products were cloned into pDONR221 using BP clonase, and subsequently into pDTNF and/or pDTCF vectors using LR clonase (Thermo Fisher). This set was augmented with Gateway Entry clones received from the Pathogen Functional Genomics Resource Center (J. Craig Venter Institute); for these genes, expression vectors were generated by Gateway cloning into pDNTF and pDTCF expression vectors [28]. Plasmids were electroporated into *M. tuberculosis* H37Rv [29]. Recombinant strains were grown in liquid medium and arrayed into 96-well plates.

### Evaluation of library growth

Growth was measured in 96-well plates as follows; 10 µl of culture was inoculated into 90 µl 7H9-OADC-Tw-Hyg100 ± ATc in a 96-well plate and incubated standing at 37°C for 7 days. Growth was measured at OD_590_ and the growth ratio of induced to uninduced was calculated.

### Determination of plasmid stability

Cultures were passaged three times in 96-well plates by inoculating 10 µl into 90 µl 7H9-OADC-Tw plus Hyg and ATc for 7 days. Cell lysates were generated from 96-well plates. Plates were placed on a heating block at 100°C for 10 min; cultures were filtered through a 96-well, 0.2 µm filter plate (Millipore) by centrifugation at 4000 rpm and collected into fresh 96-well plates. PCR amplification of the gene inserts was carried out using primers Walk-F1: 5ʹ GTGAGAAGGGTCTCTGACGAG 3ʹ and pDTNF-R3: 5ʹ CCTCGAGGTCGACGGTATCG 3ʹ. Control PCR using primers to amplify the *hyg* gene was carried out using primers HygF2: 5ʹ GAACTGCGCCAGTTCCTCCG 3ʹ and HygR2: 5ʹ CTGACCGGGAACACCGTGCTC 3ʹ.

### Determination of overexpression

Ten strains were selected at random from each library plate and inoculated to an OD_590_ of 0.05 in 5 ml 7H9-Tw-OADC plus Hyg and ATc (where indicated) in three 16 mm borosilicate tubes containing stirrer bars. Growth was monitored for 7 days by OD_590_. The three cultures were pooled and bacteria harvested by centrifugation. Bacterial pellets were washed in 10 mM Tris pH 8.0, 0.05% Tween 80 and resuspended in 10 mM Tris pH 8.0. Cell-free extracts were generated by lysis using lysing matrix B and a Fastprep (MP Biomedical) at speed 6.0 for 30 s. The lysate was centrifuged for 5 min and passed through a 0.20 μm filter. For Western analysis, 60 μg of protein was loaded and electrophoresed on a 4–12% Bis-Tris gradient gel (Invitrogen) and transferred to PVDF membrane using the iBlot system (Invitrogen). Proteins were detected using an anti-FLAG rabbit IgG primary antibody (Genscript), HRP conjugated goat anti-rabbit IgG secondary antibody and detected using SuperSignal™ West Femto Maximum Sensitivity Substrate (Thermo-Fisher). An amino-terminal FLAG-BAP (Sigma-Aldrich) fusion protein was used as the positive control.

To determine mRNA levels, total RNA was extracted as described [30]. cDNA was synthesized using the Transcriptor High Fidelity cDNA Synthesis Kit (Roche) and random hexamers. Quantitative PCR was carried out with primer/probe combinations for each gene (Supplementary Table S1) on a Roche LightCycler 480 in duplicate. Samples were also evaluated in duplicate for s*igA* expression. Reactions were formulated in LightCycler 480 Multiwell 384 well plates (Roche) containing 10 μl 2x LightCycler 480 Probes Master (Roche), 7.2 μl of 5 μM primer mix, 0.2 μl of 25 μM probe, 2 μl of cDNA, and 0.6 μl PCR grade water. Samples were cycled as follows; 95°C for 10 min initial denaturation, 45 cycles of 95°C for 10 s denaturation, 56°C for 1 mi amplification, and 72°C for 1 s extension. To determine copy number, genomic DNA was used to generate a standard curve.

### Library screen

*M. tuberculosis* clones were cultured in in 100 μl 7H9-Tw-OADC-Hyg in 96-well plates for 8 days. Clones were sub-cultured into 96-well plates containing 100 μl 7H9-Tw-OADC ± ATc and incubated for 6 days (mid-log phase). Assay plates were inoculated with 10 μl culture into medium ± ATc, ± 150 μM DCS (four combinations). Plates were incubated for 5 days and growth measured at OD_590_. The growth ratio was calculated from the OD_590_ at Day 5 compared to Day 0.

### Determination of minimum inhibitory concentration

Minimum inhibitory concentrations (MICs) were determined in liquid culture by measuring OD_590_ as described [31]. Briefly, a 2-fold dilution series of compound was prepared in medium in 96-well plates. Each plate included controls for background (medium/DMSO only, no bacterial cells), zero growth (100 μM rifampicin) and maximum growth (2% DMSO), as well as a rifampicin dose response curve. Cultures were inoculated to an OD_590_ of 0.05 and incubated for 5 days before measuring growth. A dose response curve was plotted as % growth and fitted to the Gompertz model using GraphPad Prism 6. MIC was defined as the minimum concentration at which growth was completely inhibited and was calculated from the inflection point of the fitted curve to the lower asymptote (zero growth).

## Results

### Construction of a panel of expression plasmids for *M. tuberculosis*

**Figure 1:**
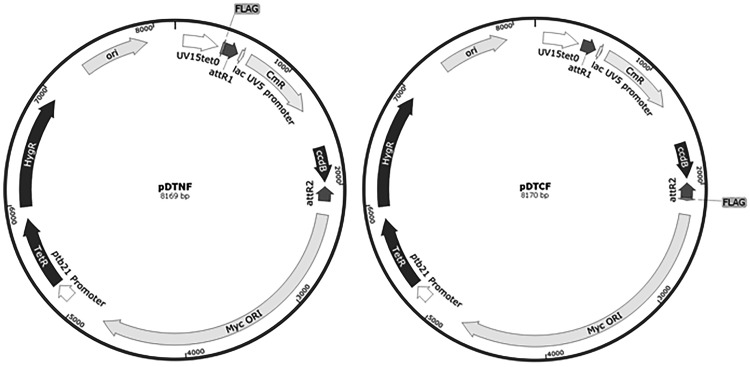
Vectors used to generate expression plasmids. pDTNF and pDTCF contain mycobacterial (MycORI) and *E.coli* replication origins (ori), hygromycin resistance, and an inducible promoter (Uv15tet0). Gene inserts are cloned into the attR1 and attR2 sites thereby eliminating the chloramphenicol and *ccdB* toxin selection markers with FLAG tags either N-terminal or C-terminal to the inserted gene.

Since overexpression of a drug target often leads to resistance, we wanted to construct a library of overexpression strains that could be used to identify the targets, or mechanisms of resistance, to novel chemical agents. Our overall aim was to use such a panel to support phenotypic screening. We aimed to construct a library of *M. tuberculosis* strains, each overexpressing a single gene (or operon in a few cases) which could be screened rapidly for growth in a high-throughput format. We decided to use an inducible expression system in order to minimize any issues arising from toxic effects of overexpression of individual genes. We used the expression plasmids pDTNF or pDTCF in which the gene is cloned downstream of a tetracycline-inducible promoter with either an N-terminal or C-terminal FLAG tag fused to the protein in a shuttle vector (Fig. 1) [28].

The *M. tuberculosis* genome has ∼4000 annotated ORFs [32]. We decided to first construct an overexpression panel containing the *in vitro* essential genes identified by Sassetti *et al.* [33].We constructed expression constructs for the majority of the essential genes. Donor plasmids were obtained either from the PFGRC library or PCR-amplified and cloned into a Gateway donor vector. Final expression plasmids were generated using Gateway cloning technology. A proportion of the essential genes (87) were cloned into both vectors to generate protein fusions with either N-terminal or C-terminal FLAG-tagged proteins. We were able to construct the majority of the expression plasmids with 542/614 essential genes represented in our set. Once we had constructed the essential set, we generated a second set of plasmids representing non-essential genes. These covered genes involved in lipid metabolism, cell wall synthesis, transcriptional/translational regulation, respiration, virulence, and efflux.

Plasmids were transformed into *M. tuberculosis* and recombinants selected on hygromycin-containing plates. Transformants for each plasmid were cultured in liquid medium and then transferred into 96-well plates and numbered (Table 1). The library was organized in several groups containing a number of plates termed POETs = Plate of Over-Expressing TB and organized into smaller panels. The first panel, (POET 21-30) contained the majority of the essential genes. Panel 2 (POET 31-36) contained expression clones from genes involved in lipid metabolism along with other pathways involved in virulence, detoxification, and adaptation. Non-essential genes involved in metabolism and respiration were arrayed in POET 37-41. The full library consists of a total of 1733 recombinant strains of *M. tuberculosis* generated from 1725 plasmids (a small number of plasmids were transformed more than once) each carrying a plasmid overexpressing a single gene or operon (Supplementary Table S2).

**Table 1:** Composition of the overexpression library

	21	22	23	24	26	27	28	29	30	31	32	33	34	35	36	37	38	39	40	41	Total
Insertion sequences and phages	2	0	1	0	0	0	3	0	1	0	0	0	1	0	0	0	0	0	0	0	8
Intermediary metabolism and respiration	31	38	45	21	31	14	14	18	29	0	3	4	4	8	11	88	85	87	88	88	707
Information pathways	17	8	10	8	25	49	42	48	20	4	0	0	0	3	6	0	0	0	0	0	240
Cell wall and cell processes	12	22	12	21	14	9	12	11	14	1	3	4	1	5	6	0	1	0	0	0	148
Conserved hypothetical	17	6	8	2	8	8	7	3	8	1	2	0	2	1	2	0	2	0	0	0	77
Virulence, detoxification, adaptation	3	6	3	1	2	1	1	3	2	33	23	35	24	29	22	0	0	0	0	0	188
Lipid metabolism	3	7	8	7	3	2	5	1	7	26	31	24	38	22	20	0	0	0	0	0	204
PE/PPE	1	0	0	1	1	2	1	0	3	0	0	0	0	0	0	0	0	0	0	0	9
Regulatory proteins	2	1	1	1	4	3	3	4	4	23	26	21	18	20	21	0	0	0	0	0	152
Total	88	88	88	62	88	88	88	88	88	88	88	88	88	88	88	88	88	87	88	88	1733

Gene classifications were taken from taken from Tuberculist (now Mycobrowser) https://mycobrowser.epfl.ch. The composition of each plate (POET 21–41) is given.

### Determination of growth kinetics for each clone

**Figure 2:**
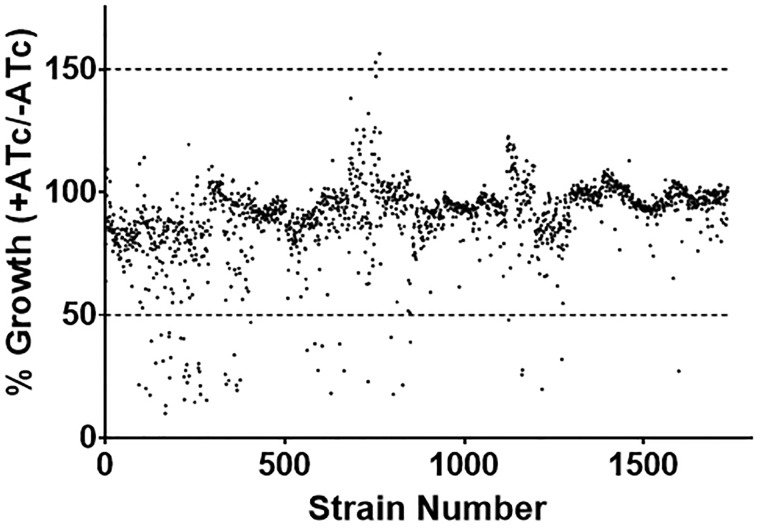
Growth of the overexpression library. The entire library was assayed for growth kinetics. Strains were inoculated using 10 µl of culture into 90 µl 7H9-OADC-Tw-Hyg100 ± ATc in a 96-well plate and incubated standing at 37°C for 7 days. Growth was measured by OD_590_. The growth ratio was calculated for each strains (OD_590_+ ATc)/OD_590_ no ATc) and expressed as a percentage of the no ATc growth.

We anticipated that overexpression of certain proteins might have a negative effect on bacterial growth; therefore we determined the growth rate for every clone in the presence and absence of ATc (induced and non-induced). Strains were cultured in 96-well plates and growth measured at Day 0 and Day 5 (Table 2 and Fig. 2). For each strain we calculated the ratio of growth under induced/uninduced conditions, since we expected that overexpression would be most likely to show a toxic effect. The majority of strains (97%) showed a growth rate comparable to the wild-type strain; using a cutoff of 1.5-fold change only 55 strains showed reduced growth (Supplementary Table S3). Only two strains showed increased growth, and that was only ∼1.5-fold over the wild-type strains (Supplementary Table S3).

**Table 2:** Growth of overexpression library strains

	96-well plate	5 ml culture
Strains evaluated	1733	155
Increased growth	2	16
Normal growth	1676	130
Reduced growth	55	9

Strains were grown in 5 ml stirred cultures or in 96-well plates ±150 ng/ml ATc for 7 days and growth measured by OD_590_. Increased growth is defined as >1.5-fold growth in the presence of ATc. Reduced growth is defined as <0.5-fold growth in the presence ATc. Normal growth is defined 0.5- to 1.5-fold growth in the presence of ATc.

### Determination of plasmid stability

A small fraction of strains had reduced growth, which we assume is due to toxicity from the expressed protein. Plasmid instability in *M. tuberculosis* has been seen, and is related to toxicity of protein expression [34]. Therefore, we expected some plasmids might be unstable due to protein toxicity [35–37], and the fact that the inducible system has leaky expression [28, 36]. We wanted to determine the stability of each plasmid over time and in multiple passages.

**Figure 3:**
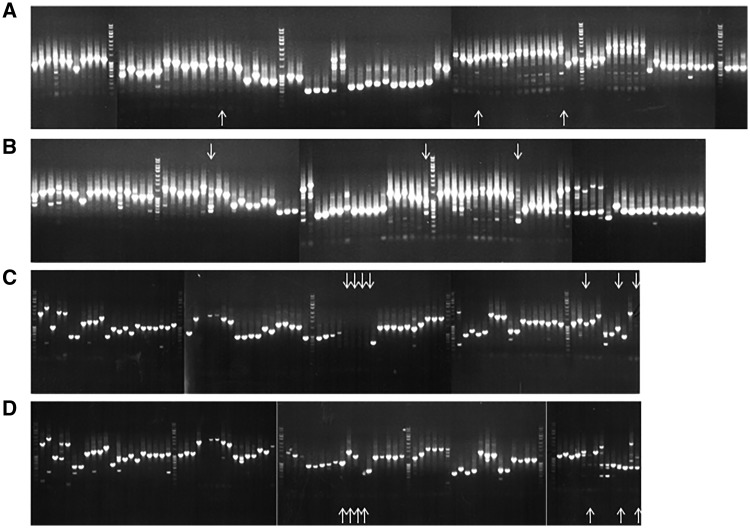
Plasmid stability in recombinant strains. *M. tuberculosis* strains were passaged three times in 96-well plates with selective pressure by inoculating 10 µl into 90 µl 7H9-OADC-Tw plus Hyg and ATc and incubating at 37°C for 7 days. Cell lysates were subjected to PCR with plasmid-specific primers designed to amplify the gene insert and insert sizes compared to the expected size. Data from two plates are presented for passage 3. (**A)** POET 32 no ATc. (**B)** POET32 + ATc. (**C)** POET34 no ATc. (**D)** POET34 +ATc. Loss or substantial reduction of expected size band observed between uninduced and induced strains are denoted by down and up arrows, respectively. Additional bands were attributed to off-target primer binding to genomic DNA within the lysate sample and not considered further.

We evaluated plasmid stability over three passages under selective pressure under both induced and uninduced conditions (Table 3 and Fig. 3). Strains were passaged three times and then tested for the presence of the correct insert size in the plasmid using primers which flanked the gene insert. Plasmids were considered stable where an amplified product of the correct size was obtained. Where no insert was amplified, we used primers for the hygromycin gene to confirm the presence of a plasmid; we were able to amplify the hygromycin resistance cassette for all these strains and they grew in the presence of hygromycin confirming that a plasmid was present. For strains in which the third passage lacked a product of the correct size, we tested the second passage, and if no correct amplicon was obtained, we tested the first passage.

**Table 3:** Stability of expression plasmids in *M. tuberculosis* recombinant strains

	No. of unstable plasmids	% Unstable
Passage 1	26	1.6%
Passage 2	43	2.7%
Passage 3	110	6.9%

A total of 1604 strains were cultured in 96-well plates and serially passaged under selective pressure. Cell lysates were generated for PCR using primers designed to amplify the gene inserts. The number of plasmids which lacked the correct size insert is given.

We evaluated the majority of the library and found that loss of the gene from the plasmid occurred only in a small subset of strains (∼7%) after three passages. After one passage, only 26 transformants (1.5%) of the entire library did not have a plasmid with the correct sized insert. Thus, we confirmed that for the majority of the library, maintenance of the plasmid even under induced conditions was stable. Of interest, the plasmids showing the most instability contained a number of essential genes, namely *embB, hycQ, ctpH, Rv1002c, rocA, hisI, pks7, folE, ftsK, fmt*, and *rplC*.

### Confirmation of protein expression in *M. tuberculosis* recombinant strains

**Figure 4:**
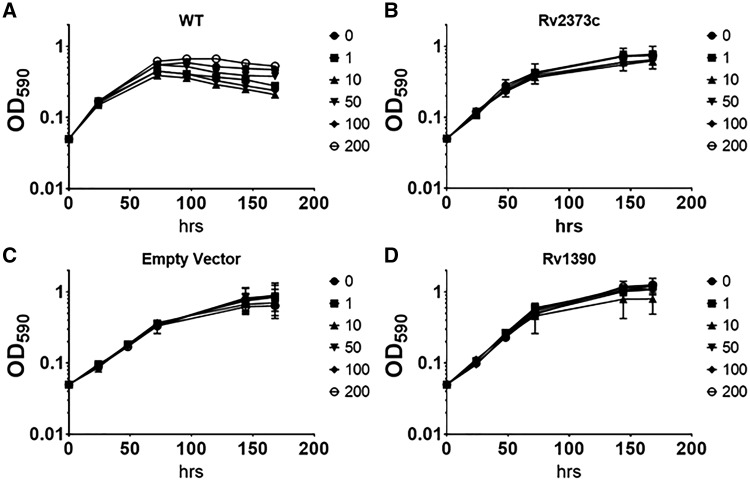
Growth of RpoZ and DNaJ2 expression strains. *M. tuberculosis* recombinant strains expressing DnaJ2 or RpoZ gene were cultured in 5 ml medium plus increasing concentrations of ATc (0–200 ng/ml). Growth was measured over time. (**A)** Wild type (no vector). (**B)** Rv2373c (DnaJ2). (**C)** Empty vector. (**D)** Rv1390 (RpoZ). Data are the mean ± SD of three independent cultures.

**Figure 5:**
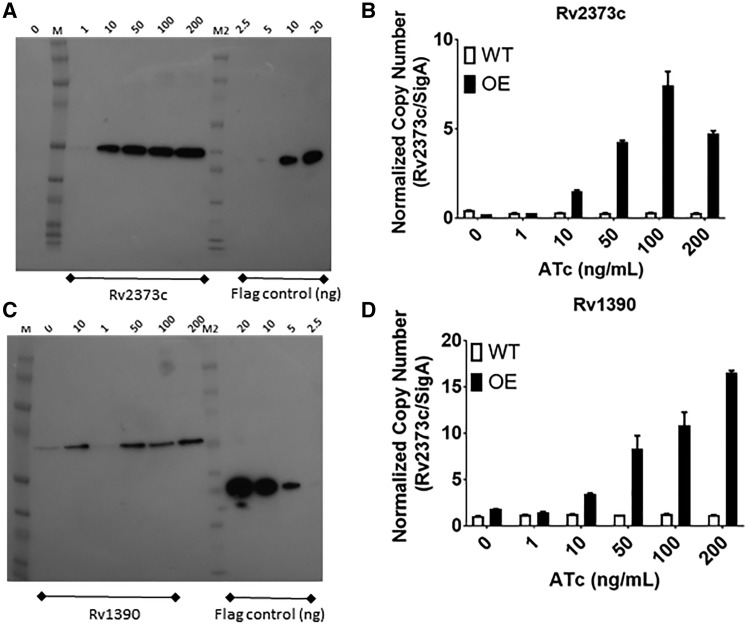
Expression of recombinant proteins in *M. tuberculosis*. *M. tuberculosis* recombinant strains expressing DnaJ2 (Rv2373c) or RpoZ (Rv1390) were cultured in 5 ml medium plus increasing concentrations of ATc (0–200 ng/ml). (**A** and **C)** Western blot using α-FLAG antibody. M = ECL Rainbow Marker; M2 = Novex Sharp Protein Standards; The amino-terminal; Flag control - FLAG-BAP fusion protein (2.5–20 ng). (**B** and **D)** RNA was isolated from cultures. cDNA was synthesized and subjected to qPCR. Copy number for each genes was determined using a standard curve generated using genomic DNA and normalized to *sigA* transcripts. Data are the mean ± SD of three independent cultures.

We wanted to confirm that expression of proteins was being maintained in an ATc-inducible fashion in our recombinant clones. We selected a subset of strains in which we determined either mRNA and/or protein levels. A subset of strains was selected randomly from the library and grown in 5 ml cultures with ATc ranging from 0 to 200 ng/ml in order to determine the expression levels in response to ATc. We also monitored growth over time for each strain. The wild-type strain showed no reduction in growth in the presence of ATc up to 200 ng/ml. The majority of strains we selected showed no significant reduction in growth up to 200 ng/ml ATc (Fig. 4). We generated cell-free extracts from these cultures and measured protein levels by Western blot using an anti-FLAG tag antibody. We also measured mRNA levels for each gene. For proteins, only the plasmid-encoded protein would be detected, whereas for mRNA we determined the total levels from both plasmid and chromosomal copies of the genes. The majority of strains demonstrated overexpression (mRNA and protein) at 100 or 200 ng/ml ATc (Table 4A). Protein expression is dependent on gene codon use, protein sequence and structure, and level of inducer; with some genes requiring high concentrations of inducer to achieve detectable levels of protein. For example, for RpoZ (Rv1390) protein expression levels plateaued at 50 ng/ml of ATc (Fig. 5C and D), whereas DnaJ2 (Rv2373c) had maximal expression at 100 ng/ml ATc and robust protein expression was observed even at 10 ng/ml ATc (Fig. 5A and B). For both of these genes, we saw inducer-dependent increases in mRNA levels (Fig. 5C and D) which were similar to protein levels, but they did not correlate exactly, either due to post-transcriptional factors or due to the fact that mRNA measures the total of native and recombinant protein.

**Table 4:** Expression from recombinant plasmids in *M. tuberculosis*

ATc (ng/ml)	Protein	mRNA
(A) Strains evaluated at variable ATc		
0	0	10
1	2	8
10	5	10
50	9	12
100	11	13
200	11	15
Total strains tested	18	17
(B) Strains evaluated at single concentration		
0	9	
150	108	
Total strains tested	396	

Recombinant strains were randomly selected from the library and evaluated for (A) mRNA and protein levels or (B) protein levels only. ATc was added at the concentrations indicated. The number of strains in which protein was detected or in which mRNA levels were increased in a dose-dependent fashion is indicated.

Once we had determined that 100–200 ng/ml was optimal for inducing protein expression, we selected a larger set of clones for evaluation. We randomly selected ∼10 strains from each plate (396 in total) and measured protein and mRNA levels. Since we saw the majority of protein expression detected at 100–200 ng/ml ATC and the strains had no growth defect, we selected 150 ng/ml ATc for subsequent work. We measured protein levels from bacteria cultured in the presence of 150 ng/ml ATc (Table 4B). We detected protein expression in 116 of the 396 strains tested by Western. Since we used cell-free extracts, we would not expect to detect membrane or secreted proteins, which applies to 53 of our clones (selected at random).

### Validation screen

We wanted to confirm that the library could be used to identify targets whose overexpression leads to resistance. To establish proof of concept, we selected DCS as our candidate drug. DCS is an established second-line antitubercular compound with known psychoactive properties [38]. DCS prevents proper cell wall construction by interfering with peptidoglycan biosynthesis; specifically the production of a mycolyl–arabinogalactan–peptidoglycan complex [39]. DCS is proposed to have two essential primary in cell targets; d-alanine racemase (Rv3423c—*alr*) and d-alanine: d-alanine ligase (Rv2981c—*ddlA*) and overexpression of these genes in *M. smegmatis* confers resistance to DCS [40, 41].

**Figure 6:**
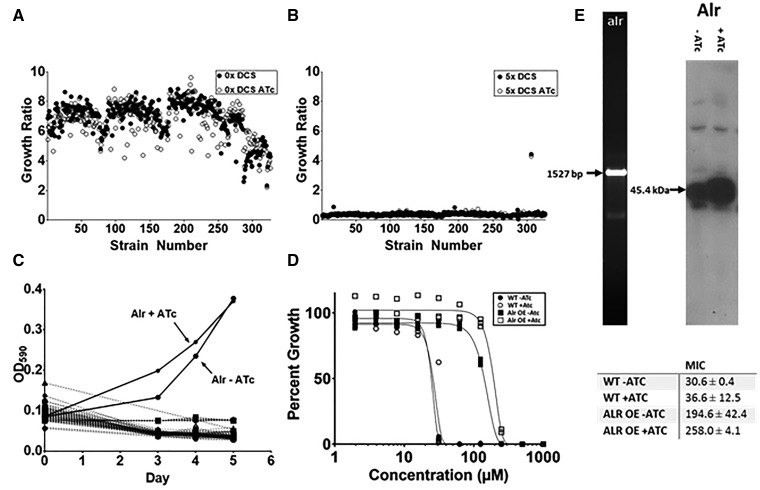
Use of the overexpression library to identify the target of a compound. *M. tuberculosis* recombinant strains were inoculated with 10 µl culture in 90 µl medium ± 150 ng/ml ATc ± 150 µM d-cycloserine. The growth ratio of each strain was calculated as (OD_590_ at Day 5)/(OD_590_ at Day 0. (**A)** No d-cycloserine. (**B)** 150 µM d-cycloserine C. (**C)** Growth kinetics of plate containing Alr-expressing recombinant strain. A single 96-well plate containing 80 independent recombinant clones was incubated for 5 days in presence of 150 µM D ± 150 ng/ml ATc. The growth kinetics for all 80 strains are shown. The Alr-expressing strain which demonstrated growth is indicated with arrows (Alt+ATc and Alr–Atc). (**D)** Determination of MICs for d-cycloserine. MICs were determined in liquid culture by measuring OD_590_ after 5 days growth at 37°C using the Gompertz algorithm [31]. (**E)** Gene integrity within the overexpression plasmid was confirmed by amplification of the gene from cell lysates using plasmid-specific primers followed by sequencing. Protein overexpression was confirmed by anti-FLAG Western.

A section of the library containing 326 clones (POET 21-24) was grown in in 96-well plates under uninduced and induced conditions and ±5X MIC (150 μM) DCS (MIC = 31 ± 0.4 μM). Growth was measured after 5 days (Fig. 6A and B). In the absence of DCS, robust growth was measured, with an average OD_590_ of 0.34 ± 0.044 (no ATc) and 0.31 ± 0.057 (plus ATc). In contrast, in the presence of DCS, minimal growth was seen, with an average OD_590_ of 0.022 ± 0.008 (no ATc) and 0.017 ± 0.006 (plus ATc). A single strain grew in the presence of DCS reaching an OD_590_ of 0.37 (plus ATc). This strain also grew in the absence of ATc induction (OD_590_ = 0.38) (Fig. 6A). The strain exhibiting robust growth contained the alanine racemase gene (Rv3423c).

We monitored the kinetics of growth in the presence of DCS for the entire 96-well plate containing the *alr* expression strain. Growth of the *alr* strain alone was observable as early as Day 3 in induced conditions. By Day 5, growth was observed for both induced and uninduced (Fig. 6B). This confirmed both that expression of Alr was leading to resistance, but also that ATc induction of expression was effective. In order to confirm resistance; we generated a fresh transformant with the *alr* expression plasmid and determined the MIC. The strain demonstrated significant resistance, with a 6–7-fold shift in MIC from the wild-type (Fig. 6D and F). The presence of the *alr* gene and overexpression of the protein was confirmed by PCR and Western, respectively confirming that protein expression was evident in both uninduced and induced states (Fig. 6E).

## Discussion

As new compound series pass through the drug development pipeline, identification of their biological target(s) and further optimization of those compound series on the target becomes essential. We have constructed a library of *M. tuberculosis* containing 1733 recombinant strains each overexpressing an individual gene or operon as a complement to existing target identification methods. The library was constructed to prioritize essential genes, as well as genes involved in cell wall and fatty acid biosynthesis, virulence factors, regulatory proteins, efflux, and aerobic and anaerobic respiration metabolic pathways; the library covers ∼40% of the protein coding genes in the *M. tuberculosis* genome [42].

The majority of the library behaved well demonstrating normal growth, stable plasmid maintenance and inducible expression of the required protein. Most of the recombinant strains tested had increased protein expression at 100–200 ng/ml of ATc which is consistent with previous studies using this expression system [28, 36]. A small number of clones were unstable, as determined by loss of the correct plasmid insert on passaging. Overexpression of these genes may be metabolically disruptive or burdensome. These genes, particularly the essential genes, may warrant further investigation as potential drug targets since modulation of their expression levels is deleterious.

When confirming the resistance of the *alr* overexpression from the screen, we noted that Alr was expressed under both the induced and uninduced conditions. Expression in the absence of inducer was also observed in a small proportion of strains evaluated by Western (9 out of 396), consistent with low level basal expression from of this ATc expression system [28, 36]. These genes (*alr, dnaJ1, hisF, argF, murD, Rv2219, acpM, topA, Rv0372c*) are all annotated as essential and it is possible that the higher expression level reflects low protein degradation and turnover rates.

We screened DCS against the overexpression library as a proof of concept; we identified *alr* overexpression strain as a target for DCS. Interestingly, the *ddlA* overexpression strains, present in the portion of the strain library screened, did not show resistance to DCS, suggesting differences between *M. smegmatis* and *M. tuberculosis.*

In conclusion, we have constructed a library of 1733 recombinant expression strains of *M. tuberculosis* for use in drug target identification and to determine the mechanisms of resistance. The library can also be used for growth on alternate media, e.g., solid medium, alternate nutrient sources, restrictive media, under different conditions, e.g., anaerobic, intra-macrophage, and will be a useful method for future drug discovery studies.

## Availability

The complete library and individual expression plasmids are available from the authors on request.

*Conflict of interest statement*. None declared.

## Supplementary Material

Supplementary Data
